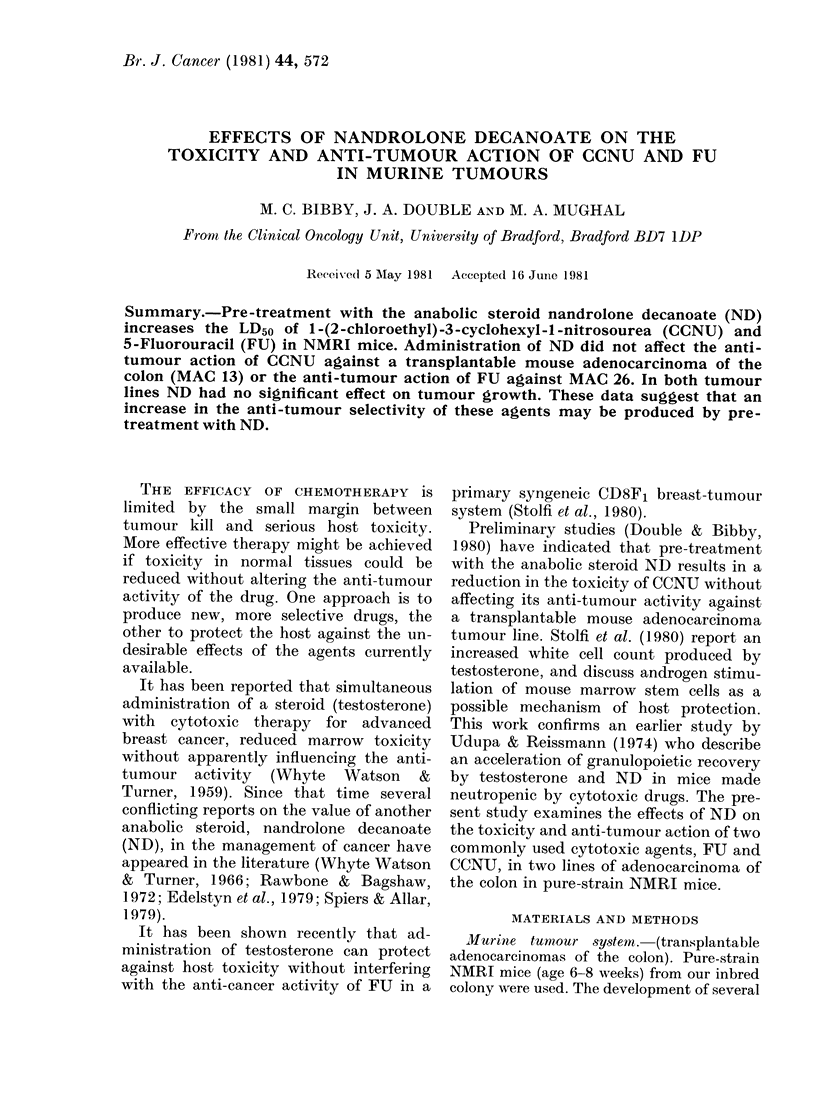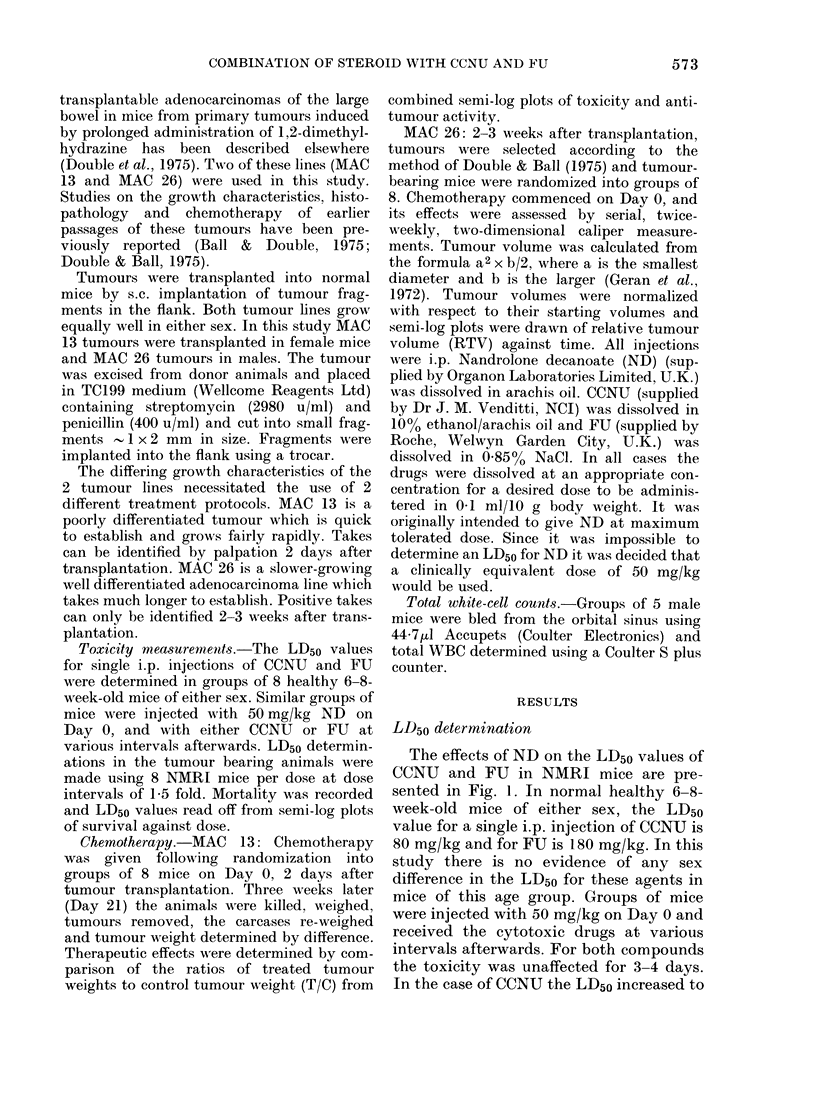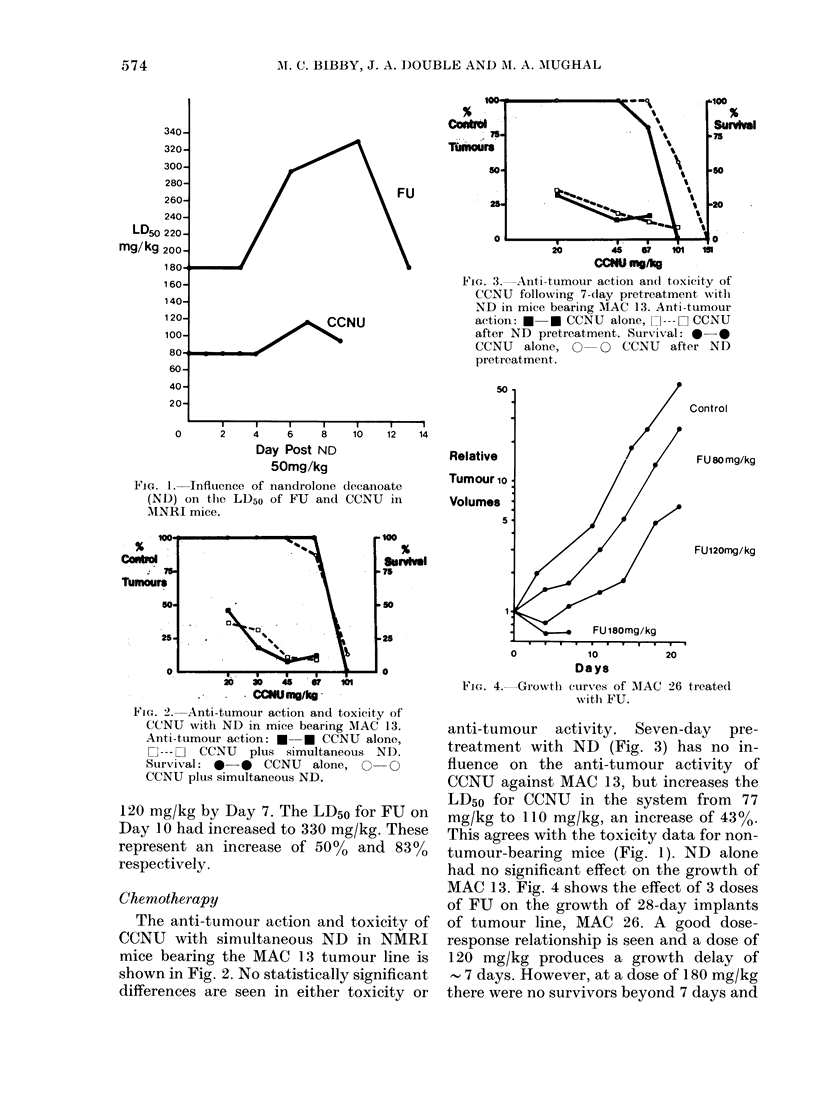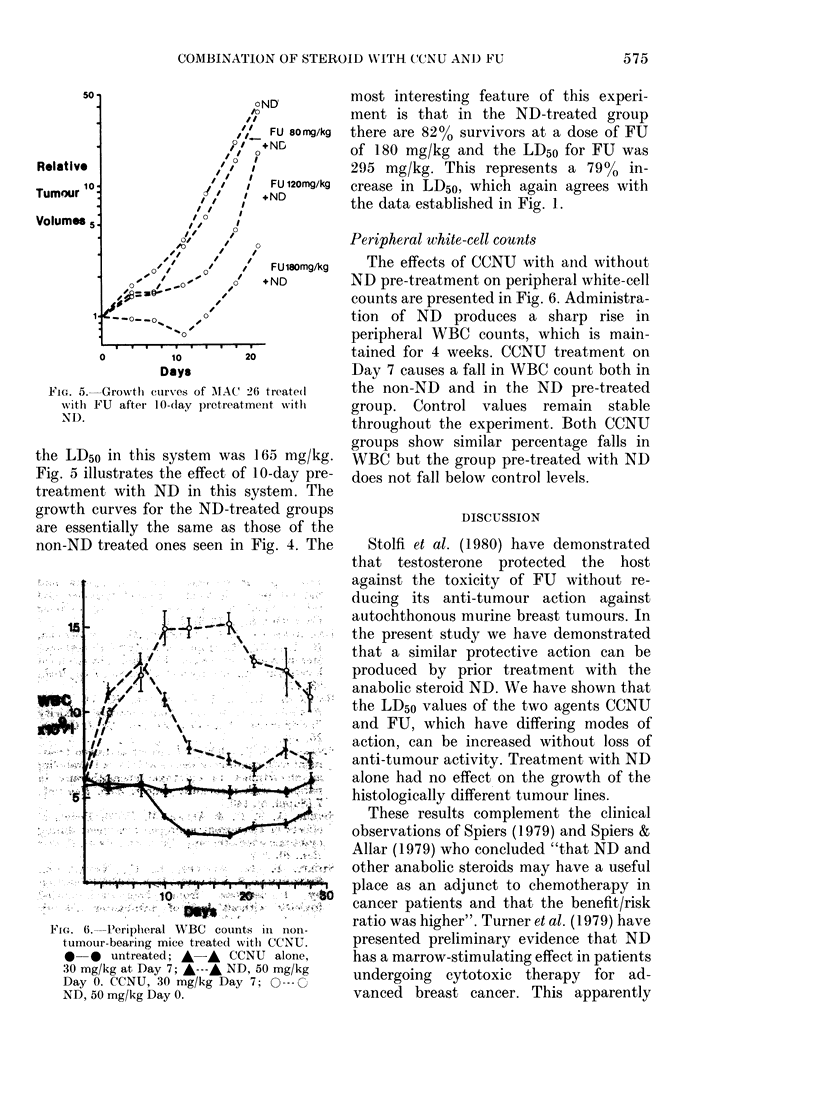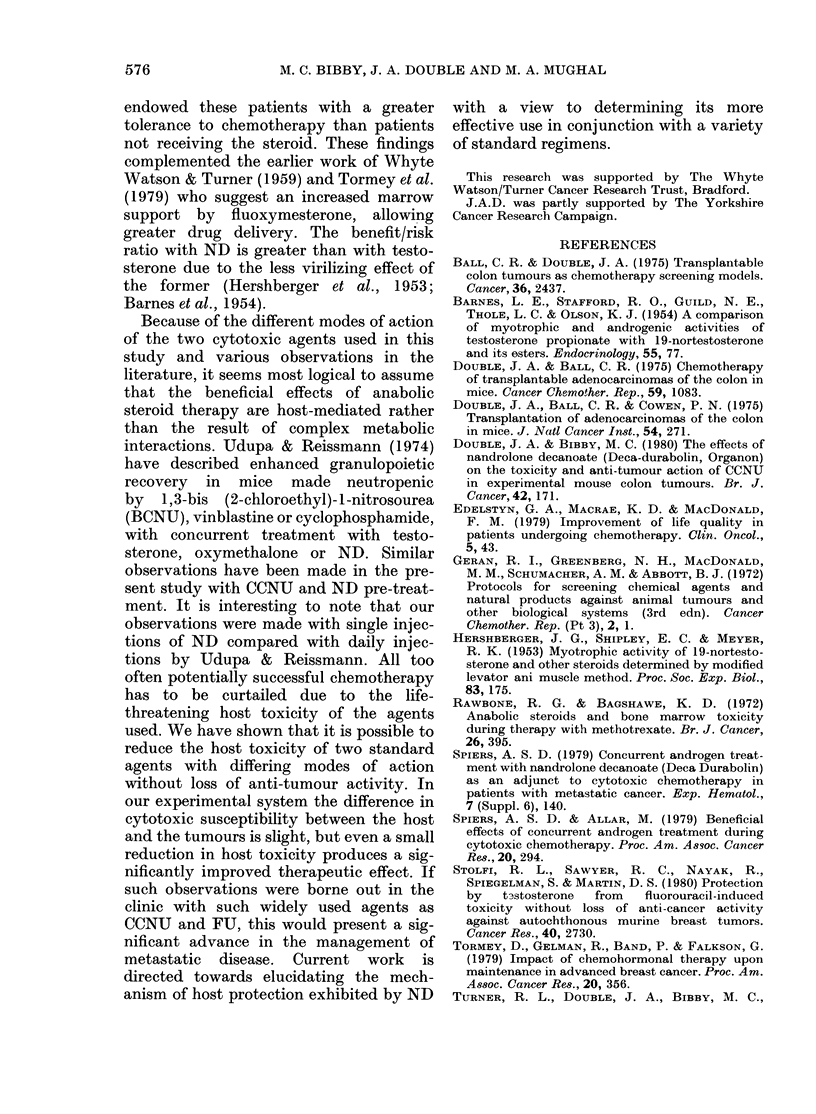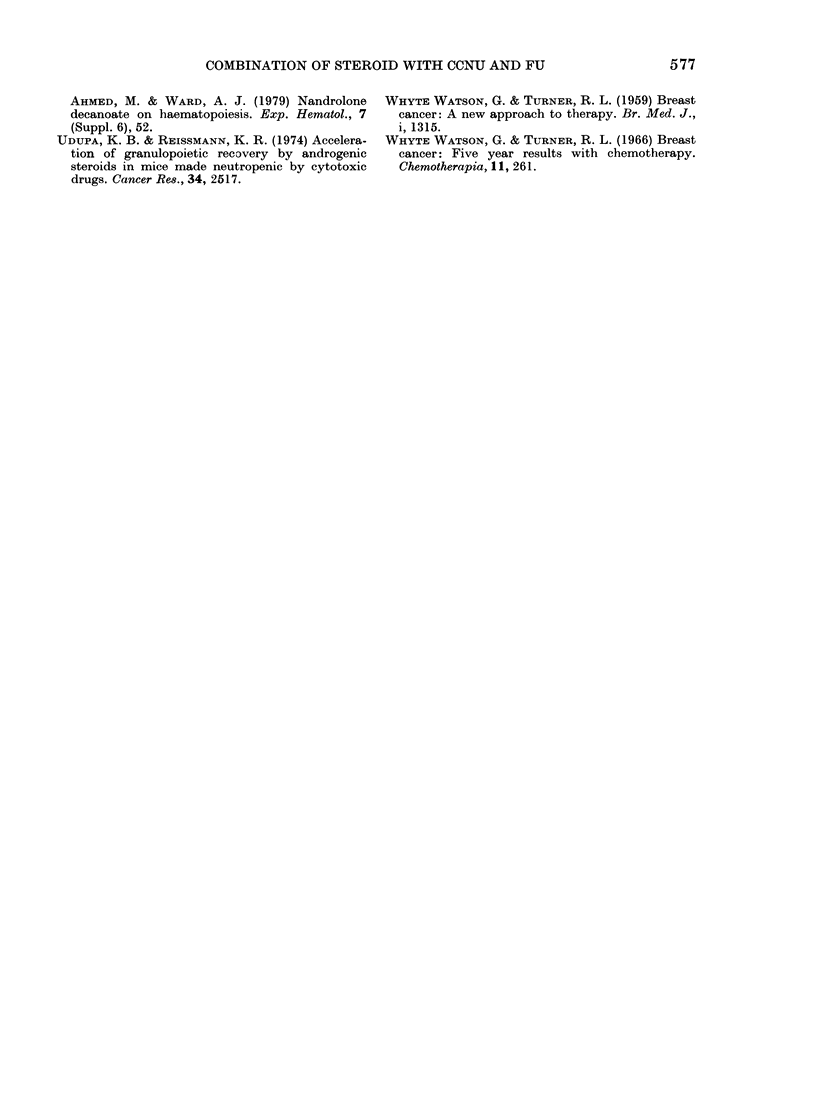# Effects of nandrolone decanoate on the toxicity and anti-tumour action of CCNU and FU in murine tumours.

**DOI:** 10.1038/bjc.1981.228

**Published:** 1981-10

**Authors:** M. C. Bibby, J. A. Double, M. A. Mughal

## Abstract

Pre-treatment with the anabolic steroid nandrolone decanoate (ND) increases the LD50 of 1-(2-chloroethyl)-3-cyclohexyl-1-nitrosourea (CCNU) and 5-Fluorouracil (FU) in NMRI mice. Administration of ND did not affect the anti-tumour action of CCNU against a transplantable mouse adenocarcinoma of the colon (MAC 13) or the anti-tumour action of FU against MAC 26. In both tumour lines ND had no significant effect on tumour growth. These data suggest that an increase in the anti-tumour selectivity of these agents may be produced by pre-treatment with ND.


*Br. J. Cancer* (1981) **44**, 572

### EFFECTS OF NANDROLONE DECANOATE ON THE TOXICITY AND ANTI-TUMOUR ACTION OF CCNU AND FU IN MURINE TUMOURS

M. C. BIBBY, J. A. DOUBLE AND M. A. MUGHAL

*From the Clinical Oncology Unit, University of Bradford, Bradford BD7 1DP*

Received 5 May 1981 Accepted 16 June 1981

**Summary.—Pre-treatment with the anabolic steroid nandrolone decanoate (ND) increases the $LD_{50}$ of 1-(2-chloroethyl)-3-cyclohexyl-1-nitrosourea (CCNU) and 5-Fluorouracil (FU) in NMRI mice. Administration of ND did not affect the anti-tumour action of CCNU against a transplantable mouse adenocarcinoma of the colon (MAC 13) or the anti-tumour action of FU against MAC 26. In both tumour lines ND had no significant effect on tumour growth. These data suggest that an increase in the anti-tumour selectivity of these agents may be produced by pre-treatment with ND.**

THE EFFICACY OF CHEMOTHERAPY is limited by the small margin between tumour kill and serious host toxicity. More effective therapy might be achieved if toxicity in normal tissues could be reduced without altering the anti-tumour activity of the drug. One approach is to produce new, more selective drugs, the other to protect the host against the undesirable effects of the agents currently available.

It has been reported that simultaneous administration of a steroid (testosterone) with cytotoxic therapy for advanced breast cancer, reduced marrow toxicity without apparently influencing the anti-tumour activity (Whyte Watson & Turner, 1959). Since that time several conflicting reports on the value of another anabolic steroid, nandrolone decanoate (ND), in the management of cancer have appeared in the literature (Whyte Watson & Turner, 1966; Rawbone & Bagshaw, 1972; Edelstyn *et al.*, 1979; Spiers & Allar, 1979).

It has been shown recently that administration of testosterone can protect against host toxicity without interfering with the anti-cancer activity of FU in a primary syngeneic $CD8F_1$ breast-tumour system (Stolfi *et al.*, 1980).

Preliminary studies (Double & Bibby, 1980) have indicated that pre-treatment with the anabolic steroid ND results in a reduction in the toxicity of CCNU without affecting its anti-tumour activity against a transplantable mouse adenocarcinoma tumour line. Stolfi *et al.* (1980) report an increased white cell count produced by testosterone, and discuss androgen stimulation of mouse marrow stem cells as a possible mechanism of host protection. This work confirms an earlier study by Udupa & Reissmann (1974) who describe an acceleration of granulopoietic recovery by testosterone and ND in mice made neutropenic by cytotoxic drugs. The present study examines the effects of ND on the toxicity and anti-tumour action of two commonly used cytotoxic agents, FU and CCNU, in two lines of adenocarcinoma of the colon in pure-strain NMRI mice.

### MATERIALS AND METHODS

*Murine tumour system.*—(transplantable adenocarcinomas of the colon). Pure-strain NMRI mice (age 6–8 weeks) from our inbred colony were used. The development of several transplantable adenocarcinomas of the large bowel in mice from primary tumours induced by prolonged administration of 1,2-dimethylhydrazine has been described elsewhere (Double *et al.*, 1975). Two of these lines (MAC 13 and MAC 26) were used in this study. Studies on the growth characteristics, histopathology and chemotherapy of earlier passages of these tumours have been previously reported (Ball & Double, 1975; Double & Ball, 1975).

Tumours were transplanted into normal mice by s.c. implantation of tumour fragments in the flank. Both tumour lines grow equally well in either sex. In this study MAC 13 tumours were transplanted in female mice and MAC 26 tumours in males. The tumour was excised from donor animals and placed in TC199 medium (Wellcome Reagents Ltd) containing streptomycin (2980 u/ml) and penicillin (400 u/ml) and cut into small fragments $\sim 1 \times 2$ mm in size. Fragments were implanted into the flank using a trocar.

The differing growth characteristics of the 2 tumour lines necessitated the use of 2 different treatment protocols. MAC 13 is a poorly differentiated tumour which is quick to establish and grows fairly rapidly. Takes can be identified by palpation 2 days after transplantation. MAC 26 is a slower-growing well differentiated adenocarcinoma line which takes much longer to establish. Positive takes can only be identified 2–3 weeks after transplantation.

*Toxicity measurements.*—The $LD_{50}$ values for single i.p. injections of CCNU and FU were determined in groups of 8 healthy 6–8-week-old mice of either sex. Similar groups of mice were injected with 50 mg/kg ND on Day 0, and with either CCNU or FU at various intervals afterwards. $LD_{50}$ determinations in the tumour bearing animals were made using 8 NMRI mice per dose at dose intervals of 1·5 fold. Mortality was recorded and $LD_{50}$ values read off from semi-log plots of survival against dose.

*Chemotherapy.*—MAC 13: Chemotherapy was given following randomization into groups of 8 mice on Day 0, 2 days after tumour transplantation. Three weeks later (Day 21) the animals were killed, weighed, tumours removed, the carcases re-weighed and tumour weight determined by difference. Therapeutic effects were determined by comparison of the ratios of treated tumour weights to control tumour weight (T/C) from combined semi-log plots of toxicity and anti-tumour activity.

MAC 26: 2–3 weeks after transplantation, tumours were selected according to the method of Double & Ball (1975) and tumour-bearing mice were randomized into groups of 8. Chemotherapy commenced on Day 0, and its effects were assessed by serial, twice-weekly, two-dimensional caliper measurements. Tumour volume was calculated from the formula $a^2 \times b/2$, where a is the smallest diameter and b is the larger (Geran *et al.*, 1972). Tumour volumes were normalized with respect to their starting volumes and semi-log plots were drawn of relative tumour volume (RTV) against time. All injections were i.p. Nandrolone decanoate (ND) (supplied by Organon Laboratories Limited, U.K.) was dissolved in arachis oil. CCNU (supplied by Dr J. M. Venditti, NCI) was dissolved in 10% ethanol/arachis oil and FU (supplied by Roche, Welwyn Garden City, U.K.) was dissolved in 0·85% NaCl. In all cases the drugs were dissolved at an appropriate concentration for a desired dose to be administered in 0·1 ml/10 g body weight. It was originally intended to give ND at maximum tolerated dose. Since it was impossible to determine an $LD_{50}$ for ND it was decided that a clinically equivalent dose of 50 mg/kg would be used.

*Total white-cell counts.*—Groups of 5 male mice were bled from the orbital sinus using 44·7 $\mu$l Accupets (Coulter Electronics) and total WBC determined using a Coulter S plus counter.

### RESULTS

### *$LD_{50}$ determination*

The effects of ND on the $LD_{50}$ values of CCNU and FU in NMRI mice are presented in Fig. 1. In normal healthy 6–8-week-old mice of either sex, the $LD_{50}$ value for a single i.p. injection of CCNU is 80 mg/kg and for FU is 180 mg/kg. In this study there is no evidence of any sex difference in the $LD_{50}$ for these agents in mice of this age group. Groups of mice were injected with 50 mg/kg on Day 0 and received the cytotoxic drugs at various intervals afterwards. For both compounds the toxicity was unaffected for 3–4 days. In the case of CCNU the $LD_{50}$ increased to

FIG. 1.—Influence of nandrolone decanoate (ND) on the $LD_{50}$ of FU and CCNU in MNRI mice.

FIG. 2.—Anti-tumour action and toxicity of CCNU with ND in mice bearing MAC 13. Anti-tumour action: ■—■ CCNU alone, □---□ CCNU plus simultaneous ND. Survival: ●—● CCNU alone, ○—○ CCNU plus simultaneous ND.

120 mg/kg by Day 7. The $LD_{50}$ for FU on Day 10 had increased to 330 mg/kg. These represent an increase of 50% and 83% respectively.

*Chemotherapy*

The anti-tumour action and toxicity of CCNU with simultaneous ND in NMRI mice bearing the MAC 13 tumour line is shown in Fig. 2. No statistically significant differences are seen in either toxicity or

FIG. 3.—Anti-tumour action and toxicity of CCNU following 7-day pretreatment with ND in mice bearing MAC 13. Anti-tumour action: ■—■ CCNU alone, □---□ CCNU after ND pretreatment. Survival: ●—● CCNU alone, ○—○ CCNU after ND pretreatment.

FIG. 4.—Growth curves of MAC 26 treated with FU.

anti-tumour activity. Seven-day pretreatment with ND (Fig. 3) has no influence on the anti-tumour activity of CCNU against MAC 13, but increases the $LD_{50}$ for CCNU in the system from 77 mg/kg to 110 mg/kg, an increase of 43%. This agrees with the toxicity data for non-tumour-bearing mice (Fig. 1). ND alone had no significant effect on the growth of MAC 13. Fig. 4 shows the effect of 3 doses of FU on the growth of 28-day implants of tumour line, MAC 26. A good dose-response relationship is seen and a dose of 120 mg/kg produces a growth delay of ~7 days. However, at a dose of 180 mg/kg there were no survivors beyond 7 days and

FIG. 5.—Growth curves of MAC 26 treated with FU after 10-day pretreatment with ND.

the $LD_{50}$ in this system was 165 mg/kg. Fig. 5 illustrates the effect of 10-day pretreatment with ND in this system. The growth curves for the ND-treated groups are essentially the same as those of the non-ND treated ones seen in Fig. 4. The most interesting feature of this experiment is that in the ND-treated group there are 82% survivors at a dose of FU of 180 mg/kg and the $LD_{50}$ for FU was 295 mg/kg. This represents a 79% increase in $LD_{50}$, which again agrees with the data established in Fig. 1.

FIG. 6.—Peripheral WBC counts in non-tumour-bearing mice treated with CCNU. ●—● untreated; ▲—▲ CCNU alone, 30 mg/kg at Day 7; ▲---▲ ND, 50 mg/kg Day 0. CCNU, 30 mg/kg Day 7; ○---○ ND, 50 mg/kg Day 0.

*Peripheral white-cell counts*

The effects of CCNU with and without ND pre-treatment on peripheral white-cell counts are presented in Fig. 6. Administration of ND produces a sharp rise in peripheral WBC counts, which is maintained for 4 weeks. CCNU treatment on Day 7 causes a fall in WBC count both in the non-ND and in the ND pre-treated group. Control values remain stable throughout the experiment. Both CCNU groups show similar percentage falls in WBC but the group pre-treated with ND does not fall below control levels.

### DISCUSSION

Stolfi *et al.* (1980) have demonstrated that testosterone protected the host against the toxicity of FU without reducing its anti-tumour action against autochthonous murine breast tumours. In the present study we have demonstrated that a similar protective action can be produced by prior treatment with the anabolic steroid ND. We have shown that the $LD_{50}$ values of the two agents CCNU and FU, which have differing modes of action, can be increased without loss of anti-tumour activity. Treatment with ND alone had no effect on the growth of the histologically different tumour lines.

These results complement the clinical observations of Spiers (1979) and Spiers & Allar (1979) who concluded "that ND and other anabolic steroids may have a useful place as an adjunct to chemotherapy in cancer patients and that the benefit/risk ratio was higher". Turner *et al.* (1979) have presented preliminary evidence that ND has a marrow-stimulating effect in patients undergoing cytotoxic therapy for advanced breast cancer. This apparently endowed these patients with a greater tolerance to chemotherapy than patients not receiving the steroid. These findings complemented the earlier work of Whyte Watson & Turner (1959) and Tormey *et al.* (1979) who suggest an increased marrow support by fluoxymesterone, allowing greater drug delivery. The benefit/risk ratio with ND is greater than with testosterone due to the less virilizing effect of the former (Hershberger *et al.*, 1953; Barnes *et al.*, 1954).

Because of the different modes of action of the two cytotoxic agents used in this study and various observations in the literature, it seems most logical to assume that the beneficial effects of anabolic steroid therapy are host-mediated rather than the result of complex metabolic interactions. Udupa & Reissmann (1974) have described enhanced granulopoietic recovery in mice made neutropenic by 1,3-bis (2-chloroethyl)-1-nitrosourea (BCNU), vinblastine or cyclophosphamide, with concurrent treatment with testosterone, oxymethalone or ND. Similar observations have been made in the present study with CCNU and ND pre-treatment. It is interesting to note that our observations were made with single injections of ND compared with daily injections by Udupa & Reissmann. All too often potentially successful chemotherapy has to be curtailed due to the life-threatening host toxicity of the agents used. We have shown that it is possible to reduce the host toxicity of two standard agents with differing modes of action without loss of anti-tumour activity. In our experimental system the difference in cytotoxic susceptibility between the host and the tumours is slight, but even a small reduction in host toxicity produces a significantly improved therapeutic effect. If such observations were borne out in the clinic with such widely used agents as CCNU and FU, this would present a significant advance in the management of metastatic disease. Current work is directed towards elucidating the mechanism of host protection exhibited by ND with a view to determining its more effective use in conjunction with a variety of standard regimens.

This research was supported by The Whyte Watson/Turner Cancer Research Trust, Bradford.

J.A.D. was partly supported by The Yorkshire Cancer Research Campaign.

### REFERENCES

BALL, C. R. & DOUBLE, J. A. (1975) Transplantable colon tumours as chemotherapy screening models. *Cancer*, **36**, 2437.

BARNES, L. E., STAFFORD, R. O., GUILD, N. E., THOLE, L. C. & OLSON, K. J. (1954) A comparison of myotrophic and androgenic activities of testosterone propionate with 19-nortestosterone and its esters. *Endocrinology*, **55**, 77.

DOUBLE, J. A. & BALL, C. R. (1975) Chemotherapy of transplantable adenocarcinomas of the colon in mice. *Cancer Chemother. Rep.*, **59**, 1083.

DOUBLE, J. A., BALL, C. R. & COWEN, P. N. (1975) Transplantation of adenocarcinomas of the colon in mice. *J. Natl Cancer Inst.*, **54**, 271.

DOUBLE, J. A. & BIBBY, M. C. (1980) The effects of nandrolone decanoate (Deca-durabolin, Organon) on the toxicity and anti-tumour action of CCNU in experimental mouse colon tumours. *Br. J. Cancer*, **42**, 171.

EDELSTYN, G. A., MACRAE, K. D. & MACDONALD, F. M. (1979) Improvement of life quality in patients undergoing chemotherapy. *Clin. Oncol.*, **5**, 43.

GERAN, R. I., GREENBERG, N. H., MACDONALD, M. M., SCHUMACHER, A. M. & ABBOTT, B. J. (1972) Protocols for screening chemical agents and natural products against animal tumours and other biological systems (3rd edn). *Cancer Chemother. Rep.* (Pt 3), **2**, 1.

HERSHBERGER, J. G., SHIPLEY, E. C. & MEYER, R. K. (1953) Myotrophic activity of 19-nortestosterone and other steroids determined by modified levator ani muscle method. *Proc. Soc. Exp. Biol.*, **83**, 175.

RAWBONE, R. G. & BAGSHAWE, K. D. (1972) Anabolic steroids and bone marrow toxicity during therapy with methotrexate. *Br. J. Cancer*, **26**, 395.

SPIERS, A. S. D. (1979) Concurrent androgen treatment with nandrolone decanoate (Deca Durabolin) as an adjunct to cytotoxic chemotherapy in patients with metastatic cancer. *Exp. Hematol.*, **7** (Suppl. 6), 140.

SPIERS, A. S. D. & ALLAR, M. (1979) Beneficial effects of concurrent androgen treatment during cytotoxic chemotherapy. *Proc. Am. Assoc. Cancer Res.*, **20**, 294.

STOLFI, R. L., SAWYER, R. C., NAYAK, R., SPIEGELMAN, S. & MARTIN, D. S. (1980) Protection by testosterone from fluorouracil-induced toxicity without loss of anti-cancer activity against autochthonous murine breast tumors. *Cancer Res.*, **40**, 2730.

TORMEY, D., GELMAN, R., BAND, P. & FALKSON, G. (1979) Impact of chemohormonal therapy upon maintenance in advanced breast cancer. *Proc. Am. Assoc. Cancer Res.*, **20**, 356.

TURNER, R. L., DOUBLE, J. A., BIBBY, M. C.,

AHMED, M. & WARD, A. J. (1979) Nandrolone decanoate on haematopoiesis. *Exp. Hematol.*, **7** (Suppl. 6), 52.

UDUPA, K. B. & REISSMANN, K. R. (1974) Acceleration of granulopoietic recovery by androgenic steroids in mice made neutropenic by cytotoxic drugs. *Cancer Res.*, **34**, 2517.

WHYTE WATSON, G. & TURNER, R. L. (1959) Breast cancer: A new approach to therapy. *Br. Med. J.*, i, 1315.

WHYTE WATSON, G. & TURNER, R. L. (1966) Breast cancer: Five year results with chemotherapy. *Chemotherapia*, **11**, 261.